# Medical Appointment—Prof. Armor

**Published:** 1857-08

**Authors:** 


### Prof. Armor.

By reference to the pages of our exchanges, we perceive with feelings of regret that the gentleman whose name stands at the head of this article has thrown off his professional robes and retired to private life. What may have been the motive for this step, wholly unlooked for by us, we cannot divine, but this much we are assured of: that is, that his own individual interests have demanded his undivided attention.

Prof. Armor was for a long time connected with our State institution, and was in fact the choice spirit in its early organization. He was loved by the classes who assembled in these halls, and is still remembered with the warmest affection and kindest regard. To his co-laborers in the Faculty he was gentlemanly, courteous and kind. No one could have manifested a greater disposition to promote the interests of the institution with which he was then connected; no one ever possessed more ability in the maintenance of its standing, nor contributed more to its efficiency or usefulness. His retirement was regretted by all, and we are assured that the step was then taken because he conceived his own best interests demanded the acceptance of one of the pressing invitations to remove elsewhere, and not from any indisposition toward a majority of the Faculty, then his colleagues, or any of those who co-operated with him then, and who still cling to its fortunes.

Dr. Armor is one among the best lecturers of the day, and a most able teacher. Pleasing in his manner, and with a style most captivating, he secures the attention of the members of the class and impresses the truths of science indelibly upon their minds.

We much regret that medical education has lost so able a contributor, and the Faculty of the institution with which he was connected so valuable a member. D. L. M'G.

